# A new species of *Hepatozoon* in the common lancehead snake (*Bothrops atrox*) from the Eastern Amazonia region

**DOI:** 10.1017/S0031182025101388

**Published:** 2026-02

**Authors:** Fabiane Rocha de Paula, Amanda Maria Picelli, Glaucilene da Silva Costa, Ana Cláudia Calchi, Marcos Rogério André, Lucio André Viana, Felipe Arley Costa Pessoa

**Affiliations:** 1 Programa de Pós-Graduação em Biodiversidade e Saúde, Fundação Oswaldo Cruz, Instituto Oswaldo Cruz, IOC/Fiocruz, Rio de Janeiro, Brazil; 2 Laboratório de Ecologia de Doenças Transmissíveis da Amazônia, Instituto Leônidas e Maria Deane, Fiocruz Amazônia, Manaus, AM, Brazil; 3 Department of Biology, Villanova University, Villanova, PA, USA; 4 Departamento de Parasitologia, Universidade Federal de Minas Gerais, Belo Horizonte, MG, Brazil; 5 Laboratório Central de Saúde Pública do Estado de Rondônia, Porto Velho, RO, Brazil; 6 Vector-Borne Bioagents Laboratory, Faculdade de Ciências Agrárias e Veterinárias, Universidade Estadual Paulista (FCAV/UNESP), Jaboticabal, São Paulo, Brazil; 7 Laboratório de Estudos Morfofisiológicos e Parasitários, Departamento de Ciências Biológicas e da Saúde, Universidade Federal do Amapá, Macapá, AP, Brazil

**Keywords:** 18S rRNA, haemogregarines, haemoparasites, taxonomy, viperid snakes

## Abstract

Although venomous snakes from the family Viperidae, such as *Bothrops atrox*, are recognized for their medical importance due to snakebite accidents, few studies on parasitological aspects have been carried out with them, especially in the Amazonia region. Using morphological and molecular tools, we described a novel haemogregarine species infecting the common lancehead snake *B. atrox* from Eastern Amazonia, Brazil. *Hepatozoon atrocis* sp. nov. has mature gamonts that are morphologically distinct from those reported in the literature, which are often compact, with dispersed or encapsulated cytoplasm and chromatin. In the phylogeny recovered from the 18S rRNA gene, the *Hepatozoon atrocis* sp. nov. sequences formed a new clade, comprising a sister group to *Hepatozoon* spp. detected in other snakes, anurans, lizards and marsupials. This study reports the first *Hepatozoon* species described in the common lancehead snake. In addition, it provides a robust review of haemogregarine species infecting viperids from all over the world.

## Graphical abstract

**Figure:**
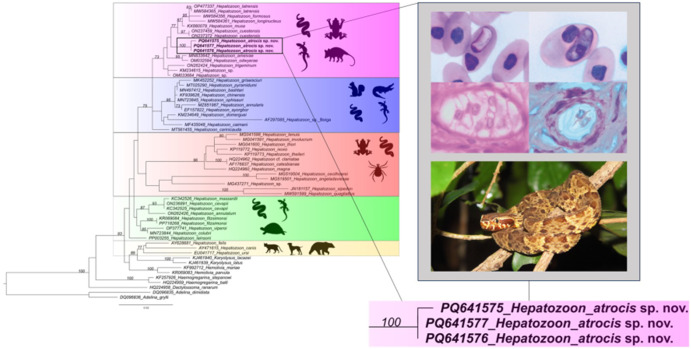

## Introduction

The genus *Hepatozoon* (Hepatozoidae) encompasses hundreds of described species, representing the most abundant and diverse haemogregarine group within the phylum Apicomplexa (Smith, 1996; Maia et al., 2016; Votýpka et al., 2017). The life cycle of these parasites is heteroxenous, involving haematophagous invertebrates as both vectors and definitive hosts, and one or more vertebrates as intermediate hosts (Votýpka et al., 2017). The range of vertebrate hosts is wide, from fish to mammals (Telford, 2009; Cardoso et al., 2022; Thomas et al., 2024). These parasites are ubiquitous in snakes, in which they can be observed as gamonts, primarily in erythrocytes, and meronts in the liver, lungs, kidneys, spleen and other viscera (Telford, 2009).

For over a century, *Hepatozoon* spp. of snakes have been described based solely on morphological and morphometric data of intraerythrocytic and tissue stages, primarily gamonts (Carini, 1910; Telford, 2009). However, morphological characterization alone may be insufficient to distinguish species and can lead to synonymy (Zechmeisterová et al., 2021). Compared to the long history of morphological descriptions, the molecular characterization of *Hepatozoon* species is still a recent development, beginning with the parasite’s characterization through the nuclear ribosomal 18S gene (Sloboda et al., 2007). Since then, many species have been described using a combination of morphological and molecular data (Maia et al., 2016; Zechmeisterová et al., 2021).

Approximately 150 species of *Hepatozoon* have been described in several snake species worldwide (Smith, 1996; Telford, 2009, 2010; Telford et al., 2012; O’Dwyer et al., 2013; Abdel-Baki et al., 2014, 2020; Han et al., 2015; Borges-Nojosa et al., 2017; Cook et al., 2018; Mansour et al., 2020; Úngari et al., 2021a, 2022; Ceylan et al., 2023; Picelli et al., 2023). Notably, the family Viperidae harbours a relatively high diversity of *Hepatozoon* spp., accounting for 25% of all described species (*n* = 38/150 spp.). These parasites have been recorded in 26 viperid snake species distributed across the Americas, Africa, Asia and Europe (Table 1). Most of these descriptions rely on morphological data; indeed, only seven species (18%; *n* = 38 spp.) have undergone molecular characterization, of which four are from two snake species, *Crotalus durissus* Linnaeus, 1758 and *Bothrops moojeni* Hoge, 1966, from southeastern Brazil (Table 1).

**Table 1.** A checklist of *Hepatozoon* species records in viperid snakes, with GenBank accession numbers, localities and references

Haemogregarine species	Snake species	GenBank	Locality	Author and year
*Hepatozoon ancistrodoni*	*Gloydius halys*	N/A	Russia	Zmeev, 1939
*Hepatozoon bashtari*	*Echis coloratus*	MN497413	Saudi Arabia	Abdel-Baki et al., 2020
*Hepatozoon bitis*^a^	*Bitis arietans*	N/A	South Africa; Uganda	Fantham, 1925; Hoare, 1932
*Hepatozoon capsulata*	*Crotalus durissus*	N/A	Colombia; Brazil	Phisalix, 1931; Pessôa, 1967a
*Hepatozoon cevapii*	*Crotalus durissus*	KC342525	Brazil	O’Dwyer et al., 2013
*Hepatozoon chodukini*	*Gloydius halys*	N/A	Uzbekistan and Kazakhstan	Markov and Bogdanov, 1961; Zechmeisterová et al., 2021
*Hepatozoon crotali*	*Crotalus atrox*	N/A	USA	Laveran, 1902; Sambon and Seligmann, 1907; Roudabush and Coatney, 1937
	*Crotalus durissus*	N/A	Brazil	Pessôa, 1967a
*Hepatozoon cuestensis*	*Crotalus durissus*	KC342527/KC342528; MF497769/MF497770	Brazil	O’Dwyer et al., 2013; Úngari et al., 2018
	*Bothrops moojeni*	ON237371/ON237372	Brazil	Úngari et al., 2022
*Hepatozoon digueti*	*Sistrurus catenatus*	N/A	Mexico	Phisalix, 1914
*Hepatozoon dogieli*	*Bitis gabonica*	N/A	Uganda	Hoare, 1920, Hoare, 1932
	*Bitis caudalis*	N/A	Namibia	Peirce and Bengis, 1998
*Hepatozoon echisi*	*Echis carinatus*	N/A	Pakistan	Mohiuddin et al., 1967
*Hepatozoon eristavi*	*Macrovipera lebetinus*	N/A	Georgia	Krasilnikov, 1964; Zechmeisterová et al., 2021
*Hepatozoon ghaffari*	*Cerastes vipera*	N/A	Egypt	Shazly et al., 1994; Abou Shafeey et al., 2019
*Hepatozoon horridus*	*Crotalus horridus*	N/A	USA	Telford et al., 2008
*Hepatozoon jararacussu*	*Bothrops jararacussu*	N/A	Brazil	Pessôa, 1968
*Haemogregarina karyolysida*^b^	*Macrovipera lebetinus*	N/A	Georgia	Krasilnikov, 1970
*Hepatozoon lebetina*	*Macrovipera lebetinus*	N/A	Algeria; Middle Asia, Caucasus	Sergent, 1918; Ovezmukhammedov, 1987; Zechmeisterová et al., 2021
*Hepatozoon massardii*	*Crotalus durissus*	KC342526	Brazil	O’Dwyer et al., 2013
*Hepatozoon mehlhorni*	*Echis carinatus*	N/A	Egypt	Bashtar et al., 1991
*Hepatozoon mocassini*	*Agkistrodon piscivorus*	N/A	USA	Laveran, 1902; Nadler and Miller, 1984; Lowichik et al., 1993; Telford, 2009
*Hepatozoon musa*	*Crotalus durissus*	MF497763–MF497766	Brazil	Úngari et al., 2018
*Hepatozoon perfilievi*	*Echis carinatus*	N/A	Turkmenistan	Zmeev, 1939
*Hepatozoon perrieri*	*Bothrops neuwiedi*	N/A	Brazil	Phisalix, 1913a
*Hepatozoon plimmeri*	*Bothrops jararaca*	N/A	Brazil	Sambon and Seligmann, 1907; Phisalix, 1913b; Pessôa, 1967b; Biase et al., 1989
	*Bothrops moojeni*	N/A	Brazil	Pessôa et al., 1971
*Hepatozoon pyramidumi*	*Echis pyramidum*	MT025290	Saudi Arabia	Mansour et al., 2020
*Hepatozoon riyadhensis*	*Cerastes gasperitii*	N/A	Saudi Arabia	Abdel-Gawad et al., 2002; Abou El–Nour et al., 2012
*Hepatozoon romani*	*Crotalus durissus*	N/A	Colombia; Brazil	Phisalix, 1931; Pessôa, 1967a
*Hepatozoon rojasi*	*Bothrops alternatus*	N/A	Argentina	Jörg, 1931
*Hepatozoon roueli*	*Bothrops alternatus*	N/A	Brazil	Phisalix and Laveran, 1913; Pessôa and De Biasi, 1972
	*Bothrops moojeni*	N/A	Brazil	Pessôa and De Biasi, 1972
*Hepatozoon samboni*	*Vipera aspis*	N/A	Italy	Sambon and Seligmann, 1907
*Hepatozoon sauritus*	*Crotalus horridus*	N/A	USA	Telford et al., 2008
*Hepatozoon seligmanni*	*Lachesis muta*	N/A	Unknown, Trinidad	Sambon and Seligmann, 1907; Everard, 1975
*Hepatozoon serpetium*^c^	*Crotalus durissus*	N/A	Brazil	Lutz, 1901
	*Bothrops* spp.	N/A	Brazil	Lutz, 1901
	*Crotalus atrox*	N/A	USA	Hilman and Strandtmann, 1960
	*Crotalus lepidus*	N/A	USA	Hilman and Strandtmann, 1960
*Hepatozoon seurati*	*Cerastes cerastes*	N/A	Algeria; Egypt	Laveran and Pettit, 1911; Foley and Catanei, 1925; Abdel-Ghaffar et al., 1991; Telford, 2009; Morsy et al., 2013
*Hepatozoon sistruri*	*Sistrurus miliarius*	N/A	USA	Telford et al., (2002)
*Haemogregarina tana*^b^	*Vipera ammodytes*	N/A	Georgia	Krasilnikov, 1970
*Hepatozoon tucumanensis*	*Bothrops alternatus*	N/A	Argentina	Senez, 1918
*Hepatozoon viperoi*	*Vipera ammodytes*	OP377741	Türkiye	Ceylan et al., 2023

a Considered a synonym of *H. dogieli* by Peirce and Bengis (1998).

b Species not included in previous reviews (see Levine 1988 and Smith, 1996).

c Lutz (1901) classified haemogregarines with distinct morphologies from several snake species as belonging to this same species, it is most likely not a valid species.

The Amazonia region has the greatest diversity of snakes in Brazil, including 12 of the 34 viperid species found in the country (Nogueira et al., 2019; Costa et al., 2022). These venomous snakes are particularly noteworthy in the region due to their frequent involvement in snakebite accidents, especially the common lancehead snake *Bothrops atrox* (Linnaeus, 1758), which is considered the most medically important species in cases of human envenomation (Monteiro et al., 2020). Furthermore, despite records of 10 species of *Hepatozoon* in Brazilian viperids (Table 1), there are no reports of these parasites in Viperidae snakes from the Amazonia region until now.

In this way, our study aimed to investigate the presence of haemogregarines in the common lancehead snake from Eastern Amazonia, Brazil. Through the combination of morphological, morphometric and phylogenetic analyses, we described a new species, namely *Hepatozoon atrocis* sp. nov., infecting the blood and tissues of *B. atrox*.

## Materials and methods

### Sampling and morphological identification

One common lancehead snake (*B. atrox*) was captured in the urban area (0°00’59.17” S and 51°04’43.39” W) of the municipality of Macapá, State of Amapá, Brazil. After restraining the snake, blood samples were collected by caudal venipuncture using sterile 1 mL insulin syringes (Sykes and Klaphake, 2008). Thin blood smears were performed, air-dried, fixed with absolute methanol for 3 min, and stained with 10% Giemsa for 30 min (Hull and Camin, 1960; Telford et al., 2001). Part of the blood sampled was stored in microtubes with 96% ethanol.

To describe the parasite stages in tissues, the snake was euthanized with the intravenous anaesthetic Pentobarbital 60–100 mg kg^−1^ and monitored until death was confirmed, following the guidelines of the Animal Ethics Committee for Veterinary Medicine (Conselho Nacional de Controle de Experimentação Animal (CONCEA), 2018). The snake was deposited as voucher material in the collection of the Laboratório de Herpetologia, Universidade Federal do Amapá, Brazil (no 4010). The liver, lungs, heart, large intestine and kidneys were collected and fixed in 10% buffered formalin to prepare histological sections. Slides were cut at 5 µm and stained with haematoxylin-eosin (Paperna and Lainson, 2004).

The blood smears and histological sections were screened under a light microscope at ×100, ×400 and ×1000 magnifications. The parasitic forms were recorded with a 5.1 MP digital camera coupled to the DI-136T biological microscope. The images and measurements of the parasites were processed using Image View® Software. The morphometric characterization of the parasite stages in blood and tissues was given in micrometres (µm), and the variables, such as length, width and area of the parasite and host cell, were presented as mean, amplitude and standard deviation. Parasitemia was estimated by counting the number of parasites visualized in 2000 erythrocytes, in 20 fields of 100 erythrocytes examined (Godfrey et al., 1987).

### DNA extraction, amplification and sequencing

DNA was extracted using the BIOPUR Mini Spin DNA/RNA extraction kit (Mobius Life Science, Pinhais, Brazil). The detection of *Hepatozoon* spp. DNA by PCR (polymerase chain reaction) was performed using the primers HEP-300 (5’-ATACATGAGCAAATCTCAAC-3’) (Ujvari et al., 2004) and ER (5’-CTTGGCCTACTAGGCATTC) (Kvicerova et al., 2008) that amplified a fragment of ≈1200 base pairs (bp) of the 18S rRNA gene. For the HepF300 and ER primer set, the PCR conditions were as follows: initial denaturation at 95°C for 3 min, followed by 35 cycles of 95°C for 30 s, with an annealing temperature of 60°C for 30 s, and an extension of 72°C for 2 min; and following the cycles a final extension step of 72°C for 10 min.

The amplicon was purified using Wizard® SV Gel and PCR Clean-Up System, following the manufacturer’s protocol. The PCR product was sequenced using the BigDye™ Terminator v.3.1 Cycle Sequencing Ready Reaction Kit (Applied Biosystems, Foster City, CA, USA) and the ABI 3100 Genetic Analyzer (Applied Biosystems, Foster City, CA, USA). Considering that the obtained sequence showed multiple peaks in the electropherogram, the amplicon was cloned into pGEM-T Easy (Promega® Madison, WI, USA), following the manufacturer’s recommendations. Six clones were selected according to the blue/white colony system. Colonies with the gene fragment of interest confirmed by PCR were subjected to plasmid DNA extraction using the Wizard® Plus SV Minipreps DNA Purification Systems (Promega Madison, Wisconsin, USA). Subsequently, plasmids were sent for sequencing with the primers M13 F (5′-CGCCAGGGTTTTCCCAGTCACGAC3′) and M13 R (5′GTCATAGCTGTTTCCTGTGTGA-3′) (Lau et al., 2010) that flank the pGEM-T Easy plasmid multiple cloning site. Furthermore, a pair of internal primers (F: 5’-TTGTTGCAGTTAAAAGTCCG-3’ and R: 5’-AACCAGACAAATCACTCCAC-3’) was designed in the present study and was also used to obtain better sequencing results. Sequencing was performed as described above.

### Phylogenetic analysis

An alignment was performed to estimate phylogenetic relationships by Maximum Likelihood (ML) among the three *Hepatozoon* spp. cloned sequences obtained in this study and those available in GenBank® using BLASTn (http://www.ncbi.nlm.nih.gov/BLAST). This alignment was constructed using the MUSCLE algorithm, available in MEGA X (Kumar et al., 2016). The database included a total of 60 18S rRNA sequences. Representative sequences of the following taxa were included: *Hepatozoon* sp. (*n* = 51), *Karyolysus* sp. (*n* = 2), *Hemolivia* sp. (*n* = 2), *Dactylosoma* sp. (*n* = 1), *Haemogregarina* (*n* = 2) and *Adelina* sp. (*n* = 2), the latter used as an outgroup. In the ML method, JModelTest version 2.1.10 was used to identify the best evolutionary model (Darriba et al., 2012). Based on the Akaike information criterion, the best model for the data was TVM + I + G. The phylogeny was inferred using the PhyML 3.0 software (Guindon et al., 2009), and the reliability of the phylogenetic relationships was evaluated in 1000 replications by the statistical calculation of the bootstrapping method (Felsenstein, 1985). The pairwise distance (*p*-distance) was performed by the MEGA X software, which generated a matrix to compare the interspecific divergence among *Hepatozoon* spp. cloned sequences from the present study, *Hepatozoon* sp. (OM033664) from *Monodelphis domestica* (Wagner, 1842), *Hepatozoon* sp. (MG437271) from *Amblyomma dissimile* Koch, 1844, and *Hepatozoon* spp. sequences previously detected in Brazilian herpetofauna.

## Results

Through microscopy screening, different stages of *Hepatozoon*, such as gamonts, meronts and dizoic cysts, were detected in blood smears and tissue sections of *B. atrox*. Morphological and phylogenetic analysis supported the proposal of a new species of *Hepatozoon*.

***Species description***

*Taxonomic summary*

Phylum Apicomplexa Levine, 1970

Class Conoidasida Levine,1988

Subclass Coccidia Leuckart, 1879

Order Eucoccidiorida Léger, 1911

Suborder Adeleorina Léger, 1911

Family Hepatozoidae Wenyon, 1926.

Genus *Hepatozoon* Miller, 1908.

*Hepatozoon atrocis* sp. nov. Paula, Picelli, Viana and Pessoa

Type host: *Bothrops atrox* (Linnaeus, 1758) (Squamata: Viperidae).

Vector: Unknown.

Type locality: Josmar Pinto Highway (AP-010), Ramal São Francisco, municipality of Macapá, Amapá, Brazil (0°00’59.17” S, 51°04’43.39” W).

Site of infection: Gamonts in blood erythrocytes; meronts in liver and intestine; dizoic cyst in intestine.

Parasitemia: 28 parasites /2000 blood erythrocytes (1·4%).

Etymology: The specific epithet ‘*atrocis*’ of *Hepatozoon atrocis* sp. nov. refers to the species name of the infected host, *Bothrops atrox*. This is the first *Hepatozoon* species described in *B. atrox*.

Type material: Two blood smear slides (hapantotypes) from *B. atrox* were deposited at the Coleção de Protozoários – COLPROT, an institutional collection of the Fundação Oswaldo Cruz Rio de Janeiro, Brazil (COLPROT 1019).

DNA sequences: The 18S ribosomal gene sequences (1305, 1253 and 1259 bp) were deposited in GenBank® (accession numbers PQ641575, PQ641576 and PQ641577).

**Figure 1.**
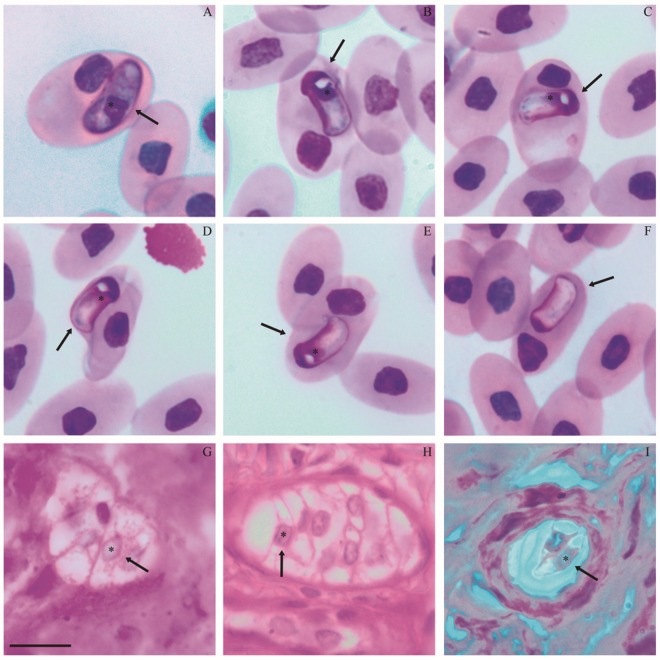
Parasitic forms of *Hepatozoon atrocis* sp. nov. in the snake *Bothrops atrox* from the Eastern Amazonia, Brazil. Blood smear with mature intraerythrocytic gamonts 1 (A) and 2 (B–F), including encapsulated forms (F). Histological sections of liver and intestine fragments with macromeronts (G, H) and dizoic cyst (I), respectively. Arrows indicate parasites. An asterisk indicates the parasite nucleus. The scale bar for all micrographs is 10 μm.

Diagnosis: Blood stages (Figure 1A–F; Table 2) – two morphotypes of mature gamonts were observed. Mature gamonts 1 (Figure 1A; Table 2). Elongated and wide, slightly arched, with rounded ends, one of which is more curved. Uniform cytoplasm stained in purple. The square nucleus with condensed dark purple chromatin is slightly displaced toward the curved end. Mature gamonts 2 (Figure 1B–F; Table 2). Compact body, arched ends, scattered cytoplasm stained in light purple in the middle of the body and dark purple at the ends. One end has a clear, rounded space, similar to a vacuole, near the nucleus. The nucleus is located near one end, with condensed or slightly dispersed chromatin, stained dark purple or pinkish purple, often without a defined shape (Figure 1D and E), and sometimes absent (encapsulated; Figure 1F). The parasitophorous vacuole is slightly evident in mature gamont 1 (Figure 1A) and not visible in mature gamont 2.

**Table 2.** Comparative morphometry of blood and tissue stages of *Hepatozoon* spp. in snakes *Bothrops* spp. from South America. Measurements are in micrometres and presented as mean ± standard deviation (SD) and ranges (minimum and maximum values)

	*Hepatozoon cuestensis*	*Hepatozoon jararacussu*	*Hepatozoon plimmeri*	*Hepatozoon perrieri*		*Hepatozoon rojasi*	*Hepatozoon roueli*		*Hepatozoon atrocis* sp. nov.	
Parasite/characteristic	O’Dwyer et al., 2013	Pessôa, 1968	Sambon and Seligmann, 1907	Phisalix, 1913a		Jörg, 1931	Phisalix and Laveran, 1913		This study	
Gamonts	*N* = 25			Mature	Immature		Mature	Immature	Mature 1 *N* = 1	Mature 2 *N* = 58
Length	14·82 ± 0·87	13–15	10–25	17–20	7–8	2–9	15–16	7–8	14·09	11·25 ± 0·42 (10·06–12·56)
Width	4·83 ± 0·92	4–5	2·5–6	2·5–5	3–4	1·5–4	2·5	2·5–3	6·1	5·38 ± 0·39 (4·73–6·81)
Area	64·62 ± 6·03								72·73	52·43 ± 3·24 (45·79–61·69)
Nucleus length	5·58 ± 0·42	6	4						4·26	5·66 ± 1·41 (2·44–9·93)
Nucleus width	3·77 ± 0·85	4	3·5–4						4·13	3·78 ± 0·81 (2·19–5·71)
Nucleus area	14·21 ± 2·04								13·08	15·12 ± 5·13 (4·95–22·76)
Infected erythrocyte									*N* = 1	*N* = 58
Length			20–22	22·5				18–22·5	22·46	20·63 ± 1·76 (15·89–24·47)
Width			10					12·5	15·06	12·55 ± 1·20 (9·31–14·8)
Area									265·8	203·70 ± 31·34 (105·91–278·39)
Nucleus length									6·67	6·91 ± 0·66 (5·83–8·25)
Nucleus width									4·9	5·21 ± 0·73 (4·14–7·01)
Nucleus area									29·1	28·51 ± 3·63 (21·24–37·53)
Uninfected erythrocytes									*N* = 30	
Length				17·5				17·5	20·78 ± 1·09 (18·11–22·78)	
Width				10				10	11·71 ± 0·94 (9·07–13·26)	
Area									197·57 ± 20·22 (134·06–226·77)	
Nucleus length									6·62 ± 0·87 (4·96–8·39)	
Nucleus width									5·09 ± 0·49 (4·09–6·19)	
Nucleus area									27·09 ± 3·16 (20·53–32·75)	
Macromeront									*N* = 10	
Meront capsule									1·64 ± ·80 (1·07–2·2)	
Length			25						31·80 ± 8·47 (19·12–41·31)	
Width			10						20·87 ± 5·37 (14·1–30·66)	
Area									540·70 ± 255·55 (158·01–926·34)	
Macromerozoites			*N* = 4						*N* = 10	
Length									14·5 ± 2·14 (11·56–18·05)	
Width									4·35 ± 0·68 (3·24–5·52)	
Area									46·89 ± 12·26 (36·42–76·33)	
Nucleus length									4·17 ± 0·53 (3·31–5·03)	
Nucleus width									3·05 ± 0·59 (2·1–4·02)	
Nucleus area									10·69 ± 2·48 (7·34–14·81)	
Micromeront										
Length		25–40	35							
Micromerozoites		*N* = 50–100	*N* = 100							
Dizoic cyst			*N* = 2						*N* = 1	
Meront capsule									3·57	
Length			25–30						14·67	
Width			10–20						11·14	
Area									120·23	
Cystozoites			*N* = 2						*N* = 2	
Length			20						10·18 ± 1·49 (9·12–11·23)	
Width									3·84 ± 0·72 (3·33–4·35)	
Area									26·55 ± 2·79 (24·57–28·52)	
Nucleus length			2–5						4·38 ± 0·76 (3·84–4·91)	
Nucleus width									2·90 ± 0·10 (2·83–2·97)	
Nucleus area									10·94 ± 1·61 (9·8–12·07)	

Tissue stages (Figure 1G–I; Table 2) – Merogony: Ten macromeronts in the liver and intestine, and 1 dizoic cyst in the intestine. Macromeronts (Figure 1G and H; Table 2). Large and wide, with a thin capsule, stained pink, sometimes ovoid or irregular in shape; containing 4–10 elongated macromerozoites with one of the ends more tapered than the other. The nuclei of macromerozoites are rounded and densely purple-stained. Dizoic cyst (Figure 1I; Table 2): ovoid, stained light pink with a well-developed capsule, containing 2 elongated cystozoites slightly tapered at both ends. The nuclei are long and rectangular, and stained light purple.

Effects on the host cell: Both mature gamonts produce noticeable effects in infected erythrocytes. Parasitized host cells were slightly hypertrophied, with their nuclei displaced to one of the extremities or margins of the host cell (Table 2).

Remarks: There are eight species of *Hepatozoon* described in *Bothrops* spp. in South America, six in Brazil, and two in Argentina (Tables 1 and 2). The first record was *Drepanidium serpentium* by Lutz in (Lutz, 1901), first renamed by Sambon (1907) as *Haemogregarina serpentium* and later renamed by Smith (1996) as *Hepatozoon serpentium* (Lutz, 1901). However, *H. serpentium* is likely not a valid species, as it was poorly described based on different gamont morphologies from several snake species, thus making any comparison with *H. atrocis* sp. nov. difficult (Lutz, 1901; Picelli et al., 2023). As the host identity and type locality for *H. serpentium* are not known and multiple species of *Hepatozoon* exist in South America, the identity of this species is unlikely to be established; therefore, we place *Hepatozoon serpentium* in the Nomen dubium here. *Hepatozoon plimmeri* (Sambon and Seligmann, 1907) from *Bothrops jararaca* (Wied, 1824) and *B. moojeni* possess claviform or rounded gamonts that are smaller and thinner or more elongated compared to *H. atrocis* sp. nov. (Phisalix, 1913b; Pessôa, 1967b; Pessôa et al., 1971) (Table 2). One macromeront in an early stage of development of *H. plimmeri* has been reported, presenting a granular mass and 4 nuclei (Phisalix, 1913b). Dizoic cysts of *H. plimmeri* were also observed, which were ovoid, like those of *H. atrocis* sp. nov., but with larger dimensions (Phisalix, 1913b) (Table 2). *Hepatozoon perrieri* (Phisalix, 1913) described in *Bothrops neuwiedi* Wagler, 1824, has young, small, ovoid gamonts that are smaller than *H. atrocis* sp. nov. (Table 2). It also has mature gamonts that are elongated, cylindrical and with rounded ends, longer and thinner than *H. atrocis* sp. nov. (Phisalix, 1913a; Table 2). *Hepatozoon jararacussu* (Pessôa, 1968) of *Bothrops jararacussu* Lacerda, 1884 has more elongated and thinner vermiform gamonts compared to *H. atrocis* sp. nov., with one rounded end and the other more tapered and curved (Pessôa, 1968; Table 2). *Hepatozoon roueli* (Phisalix and Laveran, 1913) from *Bothrops alternatus* Duméril, Bibron and Duméril, 1854 and *B. moojeni* present smaller ovoid gamonts and larger elongated and tapered vermicular gamonts than *H. atrocis* sp. nov. (Phisalix and Laveran, 1913; Pessôa and De Biasi, 1972) (Table 2). *Hepatozoon rojasi* (Jörg, 1931) and *Hepatozoon tucumanensis* (Senez, 1918) were both identified in *B. alternatus* and differ from the parasite found in the present study. Gamonts of *H. rojasi* exhibit various morphologies, which vary from elongated to short and can be cylindrical, curved, or fusiform. Their measurements demonstrate that they are smaller than the gamonts of *H. atrocis* sp. nov. (Jörg, 1931) (Table 2). Gamonts of *H. tucumanensis* are slightly curved with rounded ends; the morphometric data are unavailable for this species, thus preventing a comparison with *H. atrocis* sp. nov. (Senez, 1918). *Hepatozoon cuestensis* (O’Dwyer et al., 2013), which infects *B. moojeni*, is the only species with available molecular data and is phylogenetically related to *H. atrocis* sp. nov. Its gamonts differ from *H. atrocis* sp. nov. by being more elongated and thinner (Úngari et al., 2022) (Table 2). Four *Hepatozoon* species were phylogenetically related to *H. atrocis* sp. nov., namely *Hepatozoon musa* (Borges-Nojosa et al., 2017) from the snake *Philodryas nattereri* Steindachner, 1870; *Hepatozoon odwyerae* (Picelli et al., 2023) from the snake *Drymarchon corais* (Boie,1827); *Hepatozoon* ameivae (Carini and Rudolphi, 1912) from the lizard *Ameiva ameiva* (Linnaeus, 1758); and *Hepatozoon trigeminum* Úngari, Netherlands, Silva and O’Dwyer, 2022 from *Oxyrhopus trigeminus* Duméril, Bibron and Duméril, 1854. Gamonts of both *H. musa, H. odwyerae, H. ameivae* and *H. trigeminum* are longer and thinner (LW 18·9 ± 0·9 × 3·8 ± 0·3 μm; LW 13·41 ± 0·79 × 3·72 ± 0·35 μm; LW 14·28 ± 1·05 × 4·5 ± 0·8; LW 14·64 ± 0·58 × 5·65 ± 1·21 μm, respectively) than most gamonts of *H. atrocis* sp. nov. (Borges-Nojosa et al., 2017; Picelli et al., 2020, 2023; Úngari et al., 2022) (Table 2). In tissue stages, *H. trigeminum* had small and ovoid macromeronts (LW 19·19 ± 1·87 × 21·25 ± 2·32 μm), and elongated macromerozoites with similar dimensions (LW 15·30 ± 0·79 × 4·42 ± 0·22 μm) (Úngari et al., 2022) to those of *H. atrocis* sp. nov. (Table 2).

### Molecular and phylogenetic analysis

Three 18S rRNA cloned sequences of *H. atrocis* sp. nov. were obtained from the single individual of *B. atrox* and presented fragments of 1305 (PQ641575), 1253 (PQ641576) and 1259 (PQ641577) bp. One (PQ641575) of the three novel sequences differed by 0·2% when compared to the other two cloned sequences (PQ641576 and PQ641577), thus indicating the presence of two haplotypes (H1 [PQ641575] and H2 [PQ641576 and PQ641577]) (Supplementary Material 1).

The sequences detected in *B. atrox* exhibited BLASTn identity of 98·93–99·05% with *Hepatozoon* sp. (OM033664) from the grey short-tailed opossum, *M. domestica* (Weck et al., 2022). They also had 98·63–98·79% identity with *Hepatozoon* sp. (KM234615) from the house gecko, *Hemidactylus mabouia* (Moreau de Jonnés, 1818), and 98·71–98·86% identity with *H. musa* (KX880079) from *P. nattereri* (Harris et al., 2015; Úngari et al., 2021a). The genetic divergence between the 2 *H. atrocis* sp. nov. haplotypes and the other sequences ranged from 1 to 1·2% with *Hepatozoon* sp. (OM033664) from *M. domestica*, and 5–5·3% with *Hepatozoon quagliattus* Úngari, Netherlands, Silva and O’Dwyer, 2021 (MW591599) from the snake *Dipsas mikanii* Schlegel, 1837 (Úngari et al., 2021a) (Table 3).

**Table 3.** The pairwise distance (*p*-distance) between the haplotype sequences of *Hepatozoon atrocis* sp. nov. from the present study with *Hepatozoon* spp. sequences obtained from a marsupial, tick and herpetofauna of Brazil (1397 bp)

		1	2	3	4	5	6	7	8	9	10	11	12	13	14	15	16	17	18	19	20	21
1	PQ641575 *H. atrocis* sp. nov. H1																					
2	PQ641576 *H. atrocis* sp. nov. H2	0·002																				
3	PQ641577 *H. atrocis* sp. nov. H2	0·002	0·000																			
4	KX880079 *H. musa*	0·013	0·011	0·011																		
5	MF435048 *H. caimani*	0·021	0·020	0·020	0·017																	
6	MW584356 *H. formosus*	0·017	0·015	0·015	0·007	0·018																
7	MW584365 *H. latrensis*	0·014	0·013	0·013	0·004	0·015	0·005															
8	MW591599 *H. quagliattus*	0·053	0·050	0·050	0·058	0·055	0·060	0·058														
9	OM033664 *H.* sp.	0·012	0·010	0·010	0·010	0·014	0·014	0·012	0·051													
10	ON237372 *H. cuestensis*	0·015	0·013	0·013	0·006	0·019	0·012	0·009	0·056	0·012												
11	ON237459 *H. cuestensis*	0·014	0·013	0·013	0·006	0·017	0·012	0·009	0·054	0·011	0·000											
12	ON2624261 *H. annulatum*	0·027	0·025	0·026	0·025	0·021	0·029	0·028	0·059	0·020	0·025	0·025										
13	OP477337 *H. latrensis*	0·014	0·013	0·013	0·004	0·015	0·005	0·000	0·057	0·012	0·009	0·008	0·028									
14	PP003255 *H. lainsoni*	0·029	0·027	0·027	0·027	0·019	0·031	0·027	0·053	0·020	0·027	0·026	0·016	0·027								
15	KC342526 *H. massardii*	0·035	0·037	0·037	0·037	0·030	0·043	0·036	0·070	0·025	0·034	0·029	0·008	0·036	0·024							
16	KC342525 *H. cevapii*	0·033	0·035	0·035	0·035	0·028	0·041	0·034	0·068	0·023	0·032	0·027	0·006	0·034	0·022	0·002						
17	ON236891 *H. cevapii*	0·023	0·021	0·022	0·017	0·019	0·022	0·019	0·055	0·017	0·017	0·016	0·010	0·019	0·018	0·002	0·000					
18	MW584361 *H. longinucleus*	0·016	0·014	0·014	0·008	0·020	0·009	0·006	0·060	0·014	0·013	0·012	0·032	0·006	0·030	0·040	0·038	0·024				
19	MN833642 *H. ameivae*	0·017	0·016	0·016	0·013	0·025	0·021	0·014	0·061	0·012	0·013	0·012	0·030	0·013	0·030	0·032	0·030	0·030	0·015			
20	OM032594 *H. odwyerae*	0·019	0·017	0·017	0·016	0·031	0·021	0·018	0·068	0·016	0·016	0·016	0·035	0·017	0·037	0·038	0·036	0·037	0·019	0·004		
21	MT561455 *H. carinicauda*	0·025	0·025	0·025	0·022	0·020	0·028	0·022	0·063	0·017	0·023	0·020	0·027	0·022	0·026	0·026	0·024	0·027	0·026	0·019	0·022	
22	MG437271 *H.* sp.	0·030	0·028	0·028	0·031	0·025	0·033	0·033	0·065	0·025	0·031	0·031	0·020	0·034	0·015	n/c	n/c	0·024	0·039	n/c	n/c	n/c

**Figure 2.**
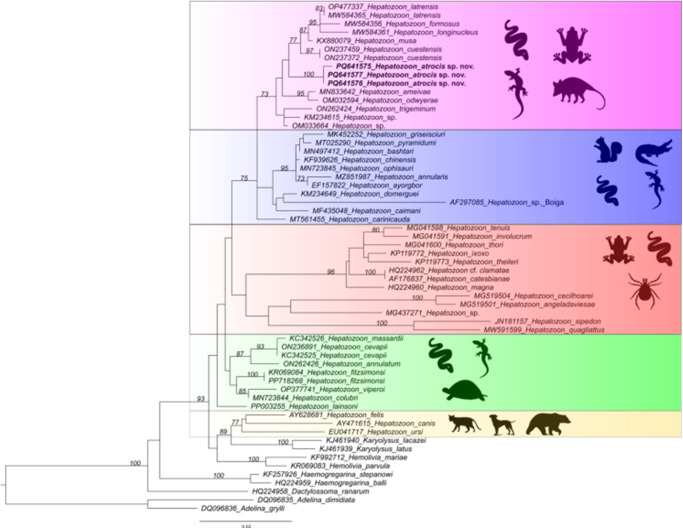
Consensus phylogenetic tree using the maximum likelihood (ML) method, based on partial sequences of the 18S rRNA gene of *Hepatozoon atrocis* sp. nov. obtained in the present study (highlighted in bold) and sequences deposited in the GenBank database (1397 bp). Accession numbers are indicated in the sequences.

The ML phylogenetic analysis resulted in a tree of 1397 bp alignment (Figure 2). The phylogenetic analysis included isolates of haemogregarines (*Haemogregarina, Hepatozoon, Karyolysus, Hemolivia* and *Dactylosoma*), and coccidia (*Adelina*) as an outgroup. The topology showed that the new haplotype sequences of *H. atrocis* sp. nov. were recovered into a well-supported small clade (bootstrap: 100%) within a larger clade comprising *Hepatozoon* sequences derived from anurans, squamates and marsupials sampled in Brazil (Figure 2). The sequences obtained from other snake hosts that shared the same ancestor with *H. atrocis* sp. nov. were the following: *H. cuestensis* (ON237459 and ON237372) from *Leptodeira annulata* (Linnaeus, 1758) and *B. moojeni*, respectively; *H. musa* (KX880079) from *P. nattereri; H. odwyerae* (OM032594) from *D. corais*; and *H. trigeminum* (ON262424) from *O. trigeminus* (Figure 2).

## Discussion

To our knowledge, we presented the first description of a haemogregarine infecting a viperid snake from the Amazonia region. Viperidae is one of the most diverse snake families, comprising approximately 400 species distributed across Europe, Africa, Asia and the Americas, including oceanic islands (Alencar et al., 2016). These venomous reptiles can be found in a wide range of ecosystems and are often abundant in their habitats (Maritz et al., 2016). Given such characteristics, viperids are compelling models for investigating *Hepatozoon* spp. diversity. The high diversity of species of *Hepatozoon* (*n* = 38) recorded in a small number of host species (*n* = 26) within the viperid taxon further supports the potential of viperids for such studies (see Table 1). On the other hand, these numbers also demonstrate our lack of knowledge about the parasitism of haemogregarines in this snake family – e.g., less than 10% of the viperid species have been reported as hosts for *Hepatozoon* spp. Besides that, there is a lack of molecular data for most of these described species to compare them in an accurate phylogenetic context.

Although the common lancehead snake is widely distributed in northern South America (Nogueira et al., 2019), *Hepatozoon* spp. have been detected in this host only in French Guiana and Brazil, albeit without species description (Thoisy et al., 2000; Kindlovits et al., 2017; Ogrzewalska et al., 2019). Although there are records of three *B. atrox* snakes positive for *Hepatozoon* spp. in French Guiana, the authors provide neither morphological nor molecular data, thus making comparison impossible (Thoisy et al., 2000). In Brazil, *Hepatozoon* sp. DNA was detected in *A. dissimile* ticks collected from *B. atrox*, which was not investigated for haemoparasite infections (Ogrzewalska et al., 2019). This sequence (MG437271) did not show identity to the sequences in our study and exhibited high similarity to *Hepatozoon* spp. detected in mammals and birds (98·2%) (Ogrzewalska et al., 2019). Another study detected a haemogregarine similar to *H. atrocis* sp. nov. in one captive *B. atrox* in Rio de Janeiro, Brazil (Kindlovits et al., 2017). Still, the absence of morphometric and molecular data prevents confirmation.

The parasitemia observed in blood smears of *B. atrox* can be considered relatively low for haemogregarines in snakes, which in extremely high infections can reach more than 60% of infected erythrocytes (Brown et al., 2006; Telford, 2009). Data on parasitemia of *Hepatozoon* in viperids were recorded only in a few studies and ranged from 0·1% to 60% (Nadler and Miller, 1984; Peirce and Bengis, 1998; Telford et al., 2002; Morsy et al., 2013; Úngari et al., 2018, 2022; Abdel-Baki et al., 2020; Mansour et al., 2020; Ceylan et al., 2023). Low parasitemia may be related to the time of infection and/or the life stage of the host (Santos et al., 2005; Brown et al., 2006). The *B. atrox* sampled here was an immature juvenile female, measuring 65·5 cm (snout-vent length) and weighing 80 g (Silva et al., 2017). In this case, the parasitemia might be explained by vertical transmission (Kauffman et al., 2017), or due to predation of infected intermediate vertebrate hosts early in the juvenile stage (Smith, 1996; Monteiro et al., 2020). Furthermore, snakes with recent infections up to eight weeks may present very low parasitemia ranging from 0·2% to 0·3% (Lowichik et al., 1993; Telford, 2009).

Morphological comparisons between *H. atrocis* sp. nov. and other *Hepatozoon* species infecting *Bothrops* spp. snakes revealed distinct characteristics (see Remarks). While some species exhibit similar gamont size ranges, the inability to accurately differentiate between mature and immature stages precludes precise comparisons (see Table 2). Moreover, morphometry may not be a reliable taxonomic criterion, as blood stages of haemogregarines can exhibit remarkable similarity, even among distinct species (Telford, 2009). For instance, despite *H. cuestensis, Hepatozoon cevapi* (O’Dwyer et al., 2013) and *Hepatozoon massardii (*O’Dwyer et al., 2013) shared morphological features (e.g. elongated shapes) and comparable dimensions (17·07 ± 1·44 × 3·6 ± 0·55 µm, 17·05 ± 1·10 × 3·12 ± 0·49 µm and 17·31 ± 1·00 × 2·95 ± 0·38 µm; respectively), they represent different species (O’Dwyer et al., 2013; Úngari et al., 2022). Another example involves *Hepatozoon catesbianae* (Stebbins, 1904) and *Hepatozoon clamatae* (Stebbins, 1905), species with morphologically indistinguishable gamonts. Historically, these were differentiated based on cytopathological alterations seemingly specific to *H. clamatae* in anuran hosts. However, molecular data have since shown they are not distinct species (Léveillé et al., 2021). Conversely, the same species may exhibit different morphometries, as seen with *H. cevapii*, which showed distinct morphometric variations in records made by its original authors (O’Dwyer et al., 2013; Úngari et al., 2022).

Only macromeronts (first-generation meronts) were observed, producing macromerozoites, which are larger and less numerous than the micromerozoites formed by micromeronts (second-generation meronts) (Telford, 2009). The dizoic cyst represents latent stages, and it is typically found in the liver (Desser, 1993; Smith, 1996), but was present in the *B. atrox* intestine. Its presence may indicate a role in transmission through predation of another *B. atrox* (Fraga et al., 2013; Rodrigues et al., 2016). However, it is more likely that dizoic cysts provide a continuous infection for the snake, as they can eventually differentiate into meronts throughout infection (Desser, 1993; Telford, 2009).

Regarding the genetic data, we observed a 0·2% *p*-distance between the *H. atrocis* sp. nov. 18S rRNA sequences, differing in two nucleotides. Notably, similar intraspecific sequence divergence is documented in other snake-associated *Hepatozoon* species. For instance, the two sequences of *H. quagliattus* showed 100% similarity and a 0·2% *p*-distance (Úngari et al., 2022), and *H. musa* sequences with >99% similarity and a 0·2% *p*-distance (Úngari et al., 2018). Conversely, although *H. massardii* and *H. cevapii* were initially described as distinct species based on a 0·2% divergence (O’Dwyer et al., 2013; Úngari et al., 2018), these two species shared >99% identity (Úngari et al., 2018, 2022) and were shown to be closely related and positioned within the same clade (Úngari et al., 2018, 2022). Even when comparing the short sequence of *H. massardii* (unique sequence) with the longer sequences of *H. cevapii* from studies conducted after its initial description (Úngari et al., 2022). Furthermore, their morphological and morphometric characteristics are highly similar, with only minor variations in cytoplasm staining (O’Dwyer et al., 2013). Gene mutations resulting from the cloning process can cause nucleotide polymorphisms and DNA divergence (Ferreira et al., 2020). This was observed in haplotype sequences of *Hepatozoon* sp. from the anurans *Leptodactylus latrans* (Steffen, 1815) and *Rhinella diptycha* (Cope, 1862) (Ferreira et al., 2020). A recent study on *Hepatozoon caimani* (Carini, 1909) from the caiman *Caiman yacare* (Daudin, 1801) identified distinct haplotypes with polymorphisms in several nucleotides and positioned them in subclades within the phylogeny (Clemente et al., 2023). Despite this genetic variation, the authors did not consider it sufficient to determine new species designations, as all haplotypes maintained >99% identity with *H. caimani* (Clemente et al., 2023). Similarly, the observed divergence in the cloned sequences of *H. atrocis* sp. nov. likely represents intraspecific haplotype variation (H1 and H2) within the same host species rather than distinct species.

Overall, the recovered phylogeny presented herein showed the known paraphyletic pattern of the genus, with the organization of two major distinct clades separating *Hepatozoon* of carnivores from other species (Karadjian et al., 2015; Zechmeisterová et al., 2021; Thomas et al., 2024). It also maintained the phylogenetic relationships of *Hepatozoon* spp. from Brazilian snakes, which were distributed into four main clades (Paula et al., 2022; Úngari et al., 2022; Picelli et al., 2023). The 18S rRNA haplotypes of *H. atrocis* sp. nov. clustered in one of these clades, forming a sister taxon of two subclades composed of *Hepatozoon* lineages from different vertebrate groups (i.e. anurans, squamates and marsupial) mainly from the Brazilian Cerrado biome (Úngari et al., 2021b, 2022). This relationship between *Hepatozoon* species in herpetofauna and small mammals is well-established, as evidenced by phylogenetic studies on this parasite genus (Thomas et al., 2024). The proximity of *H. atrocis* sp. nov. with *Hepatozoon* species of anurans and the grey short-tailed opossum indicates a potential use of the same vector group by these parasites. Considering that several tick species that parasitize *B. atrox* snakes can also be found in anurans (Mendoza-Roldan et al., 2020) and small mammals (Guglielmone and Nava, 2010), the role of ticks as vectors of *Hepatozoon* across this clade should be further investigated (Mendoza-Roldan et al., 2021). Additionally, such phylogenetic relationships could be explained by the transmission of *Hepatozoon* via the food web (Sloboda et al., 2008; Thomas et al., 2024). The detection of *Hepatozoon* sp. in lung and spleen samples of *M. domestica* might indicate that this marsupial species may have a role as a paratenic host for snake-associated *Hepatozoon* spp. (Carvalho et al., 2019; Weck et al., 2022; Thomas et al., 2024). Furthermore, despite being considered a generalist species, *B. atrox* has ontogenetic shifts in diet and microhabitat use (Bisneto and Kaefer, 2019; Monteiro et al., 2020). While juveniles commonly occupy higher strata in the vegetation and have a diet consisting of ectothermic animals (e.g. fish, frogs, lizards and snakes), adults are typically found on the ground and prey on endothermic animals (e.g. small mammals and birds) (Monteiro et al., 2020), including marsupials (Voss, 2013).

Our phylogenetic results demonstrate that *Hepatozoon* parasites of viperids have different evolutionary origins, which are not necessarily related to host taxonomy or geographic distribution. The lineages described in Brazilian viperid snakes were positioned in four distinct clades. The new small clade contained sequences of *H. atrocis* sp. nov. that clustered closely with the *H. cuestensis* clade and related to this last one the clade of *H. musa*. Another distinct group contained *H. cevapii* and *H. massardii*, which were distant from all the other lineages. The relationships among *H. cuestensis, H. cevapii, H. massardii* and *H. musa* remained consistent with previous studies (O’Dwyer et al., 2013; Úngari et al., 2018). This pattern was also observed in Saudi Arabian viperid species, *Hepatozoon bashtari* (Abdel-Baki et al., 2020) of *Echis coloratus* Günther, 1878 (Abdel-Baki et al., 2020) and *Hepatozoon pyramidumi* (Mansour et al., 2020) of *Echis pyramidum* (Geoffroy Saint-Hilaire, 1827) (Mansour et al., 2020), which formed a distinct clade separate from other viperid species. Similarly, *Hepatozoon viperoi* (Ceylan et al., 2023) from *Vipera ammodytes* (Linnaeus, 1758) from Türkiye (Ceylan et al., 2023) also grouped distinctly but closely with *H. cevapii* and *H. massardii* from Brazil (O’Dwyer et al., 2013).

The proximity of *H. atrocis* sp. nov. and *H. cuestensis* may be explained by the shared life history of their hosts. Indeed, the *H. cuestensis* sequence was the only one, so far, obtained from another snake species of the genus *Bothrops, B. moojeni* (Úngari et al., 2022). Both *B. moojeni* and *B. atrox* are generalist predators that consume a similar range of prey, including rodents, amphibians and lizards (Nogueira et al., 2003). While *B. moojeni* is primarily found in the Cerrado and Pantanal biomes and does not overlap with *B. atrox* in our study region, these species can co-occur in some areas of Brazil (Nogueira et al., 2019; Costa et al., 2022). Thus, exposure to similar arthropod vectors or shared paratenic hosts due to overlapping ecological niches potentially facilitates the transmission of closely related *Hepatozoon* species.

A broader understanding of the relationships in the evolutionary history of *Hepatozoon* in viperid species, as well as other heteroxenous parasites, requires data on other components of their life cycle, such as the vectors (Barta et al., 2012; Votýpka et al., 2017). The natural life cycle of *Hepatozoon* in viperid snakes has not been elucidated, but through experimental infections with laboratory-reared mosquitoes, it was possible to verify the sporogonic development of at least 8 species – *Hepatozoon horridus (*Telford et al., 2008), *Hepatozoon mehlhorni* (Bashtar et al., 1991), *Hepatozoon moccassini* (Laveran, 1902), *H. plimmeri, H. roueli, Hepatozoon seurati* (Laveran and Pettit, 1911) *Hepatozoon sauritus* (Telford et al., 2001) and *Hepatozoon sistruri* (Telford et al., 2002) – indicating mosquitoes as possible vectors for these parasites (Pessôa et al., 1971; Pessôa and De Biasi, 1972; Nadler and Miller, 1984; Abdel-Ghaffar et al., 1991; Bashtar et al., 1991; Telford et al., (2002); Telford et al., 2008; Morsy et al., 2013).

In summary, morphological and morphometric characterization revealed blood and tissue stages of a haemogregarine in the common lancehead snake (*B. atrox*), which is distinct from previously described species. Molecular evidence supported the identification of a new species, namely *H. atrocis* sp. nov. This new record not only expands the geographic distribution of haemogregarines in viperids within Brazil but also provides the first genetic record of this parasite in an Amazonian viperid. In conjunction with an updated checklist of *Hepatozoon* spp. in viperids, this study offers a comprehensive view of *Hepatozoon* parasitism within this snake family, showing that this interaction is still underestimated, especially in the Amazonia region.

## Supporting information

10.1017/S0031182025101388.sm001 Rocha de Paula et al. supplementary material Rocha de Paula et al. supplementary material

## Data Availability

Sequence data are available at GenBank accessions: PQ641575, PQ641576 and PQ641577 (partial 18S rRNA). The snake specimen voucher was deposited at the Laboratório de Herpetologia of Universidade Federal do Amapá (no 4010). Parasite hapantotypes (COLPROT 1019) were deposited in the Coleção de Protozoários – COLPROT, Fundação Oswaldo Cruz – Fiocruz, Brazil.

## Supplementary material

The supplementary material for this article can be found at https://doi.org/10.1017/S0031182025101388.
